# Perspectives on the development and future of oocyte IVM in clinical practice

**DOI:** 10.1007/s10815-021-02263-5

**Published:** 2021-07-03

**Authors:** Michel De Vos, Michaël Grynberg, Tuong M. Ho, Ye Yuan, David F. Albertini, Robert B. Gilchrist

**Affiliations:** 1 Centre for Reproductive Medicine, UZ Brussel, Brussels, Belgium; 2 Department of Obstetrics, Gynecology, Perinatology and Reproductology, Institute of Professional Education, Sechenov University, Moscow, Russia; 3 Department of Reproductive Medicine and Fertility Preservation, Antoine Béclère University Hospital, Clamart, Clamart, France; 4 Paris-Sud University, Le Kremlin Bicêtre, France; 5 IVFMD, My Duc Hospital, Ho Chi Minh City, Vietnam; 6 Colorado Center for Reproductive Medicine, Lone Tree, CO 80124 USA; 7 Bedford Research Foundation, 124 South Road, Bedford, MA 01730 USA; 8 Fertility & Research Centre, School of Women’s and Children’s Health, University of New South Wales Sydney, Sydney, NSW Australia

**Keywords:** In vitro maturation (IVM), Oocyte maturation, Onco-fertility, Fertility preservation, Polycystic ovary syndrome (PCOS)

## Abstract

Oocyte in vitro maturation (IVM) is an assisted reproductive technology designed to obtain mature oocytes following culture of immature cumulus–oocyte complexes collected from antral follicles. Although IVM has been practiced for decades and is no longer considered experimental, the uptake of IVM in clinical practice is currently limited. The purpose of this review is to ensure reproductive medicine professionals understand the appropriate use of IVM drawn from the best available evidence supporting its clinical potential and safety in selected patient groups. This group of scientists and fertility specialists, with expertise in IVM in the ART laboratory and/or clinic, explore here the development of IVM towards acquisition of a non-experimental status and, in addition, critically appraise the current and future role of IVM in human ART.

## Introduction

Human ARTs (assisted reproductive technologies), while initiated to provide family building opportunities to subfertile or infertile patients, have now expanded into an important branch of contemporary medicine serving a far wider segment of the global population than anticipated following the birth of Louise Brown in 1978. From its humble beginnings, having access to human gametes and embryos has expanded the knowledge base upon which ARTs have flourished, providing for the first time the opportunity to do original research in human reproductive biology. Coming at a time of unprecedented technological breakthroughs, the ability to obtain and analyze the molecular and physiological basis of gametogenesis and embryogenesis in humans has both enriched the practice of ARTs for the benefit of patients and opened new opportunities for clinical and basic scientists alike. After many years of foundational study, in vitro maturation (IVM) has now reached a point in the history of human ARTs where its clinical utilization will both broaden and enrich the hopes of practitioners and patients in the decades ahead. It is the singular purpose of this review to provide a platform for understanding how, why, and when IVM has entered the spectrum of ARTs available to reproductive and regenerative medicine.

## Historical landmarks in the development of human IVM

**Fig. 1:**
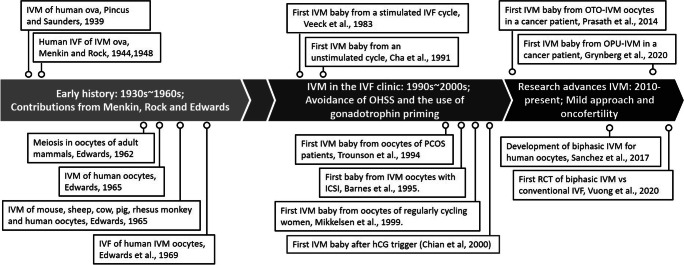
Landmarks in the development of human IVM. Major stages in the development of IVM beginning with studies using human oocytes and initial attempts at IVF and during 1990–2000 decades, first uses in the clinic, are shown. The last decade has witnessed modest expansion of clinical usage, especially in areas of onco-fertility and fertility preservation

In an ironic twist of fate, the first reports of IVF in the human were the result of IVM using ovarian oocytes. Menkin and Rock harnessed the potential of maturing oocytes in vitro based on the long-standing collaboration between Rock and Pincus. The latter first reported IVM using rabbit ova [1] and then subsequently using human oocytes [2]. Buoyed by their collaboration, Menkin and Rock reported successful fertilization of in vitro matured human ova [3, 4] (Fig. 1). In hindsight, these first reports of human IVF using IVM oocytes were notable for two reasons: they established the concept of spontaneous oocyte maturation in mammals when cumulus–oocyte masses released from Graafian follicles displayed meiotic and developmental competence over a certain time interval and, importantly, made the earliest stages of human development experimentally tractable.

Fast forward to the 1960s, Edwards made significant inroads into IVM in the human (Fig. 1) (reviewed in [5]). The kinetics and culture requirements for IVM were established for several different mammalian species [6], including for human oocytes [7]. His landmark work on human oocyte IVM established the kinetics of nuclear maturation showing metaphase 2 (MII) stage was reached at about 36 h [8]. And within a few short years, his work with Bavister led to the successful IVF of human IVM oocytes [9, 10]. The impetus to move in the direction of clinical IVF, while not to draw upon IVM as a step in the process, was realized from this pioneering work. Moreover, that mammalian oocytes from many species shared the properties implicit to spontaneous maturation provided a research platform for the next 40 years of progress in oocyte biology, maturation being the final act.

While the next 20 years (1970–1990; Fig. 1) would bring clinical IVM closer to translation, it was a pivotal period during which fundamental insights into oocyte physiology were made using animal models. The first IVM baby resulting from immature oocytes derived from oocyte donors was reported in 1991 by Cha and colleagues [11]. Since that report, IVM gained attention during the 1990s [12–14], primarily as a treatment option to reduce the risk of ovarian hyperstimulation syndrome (OHSS) associated with ovarian stimulation (OS), especially for polycystic ovary syndrome (PCOS) patients [15]. During this same time period, IVM became an experimental platform for the discovery of how the dialogue between cumulus cells and the oocyte regulate both nuclear and cytoplasmic maturation. At the heart of the debates bridging clinical and basic science for the next 20 years was the need to understand the relationship between hormonal control of oocyte maturation, its coupling to ovulation, and the molecular and cellular underpinnings of meiotic arrest and cell cycle progression to MII.

From the initial efforts of Chian, Tan, and colleagues at McGill University [16], various gonadotropin priming strategies coupled with differences in IVM protocols between clinics clouded the field [17], prompting calls for clarity regarding a simple and logical definition of oocyte IVM [18, 19]. Central to current and future efforts to bring IVM into the realm of everyday human ARTs are major research advances into the physiology of ovulation in mammals. From the earliest days implicating a single gonadotropin signal, capable of blocking the delivery of meiotic arresting factors like cGMP and cAMP, to the more recent elaboration of multiparametric signaling cascades downstream of LH reception during ovulation, the complexities and nuances emergent from 20 years of research have led to the development of a laboratory platform designed to mimic, to the best of our current capabilities, what happens naturally in vivo (reviewed in [20, 21]). Translation to the human has progressed [22, 23] due to three central physiological parameters we now have a deeper understanding of:

A. Maintenance of the cumulus–oocyte dialogue is essential to assure bioenergetic and metabolic support required to achieve cytoplasmic maturation.
B. Signaling reciprocity between oocyte-secreted factors and cumulus define temporal parameters for maturation that establish developmental competence.
C. Coordination of ovulation with meiotic maturation involves a series of metabolic and gene expression changes in somatic granulosa cells and enclosed oocytes that can now be reproducibly manipulated under in vitro conditions.

Given these extraordinary advances in reproductive physiology (as summarized below), it is imperative that IVM assumes its role in human infertility treatment, prompting the stated objective of this paper to raise the awareness from clinicians, embryologists, and patients and recognize IVM as a patient-friendly and efficient ART technique.

## The challenge: to mimic in vivo oocyte maturation in vitro

**Fig. 2:**
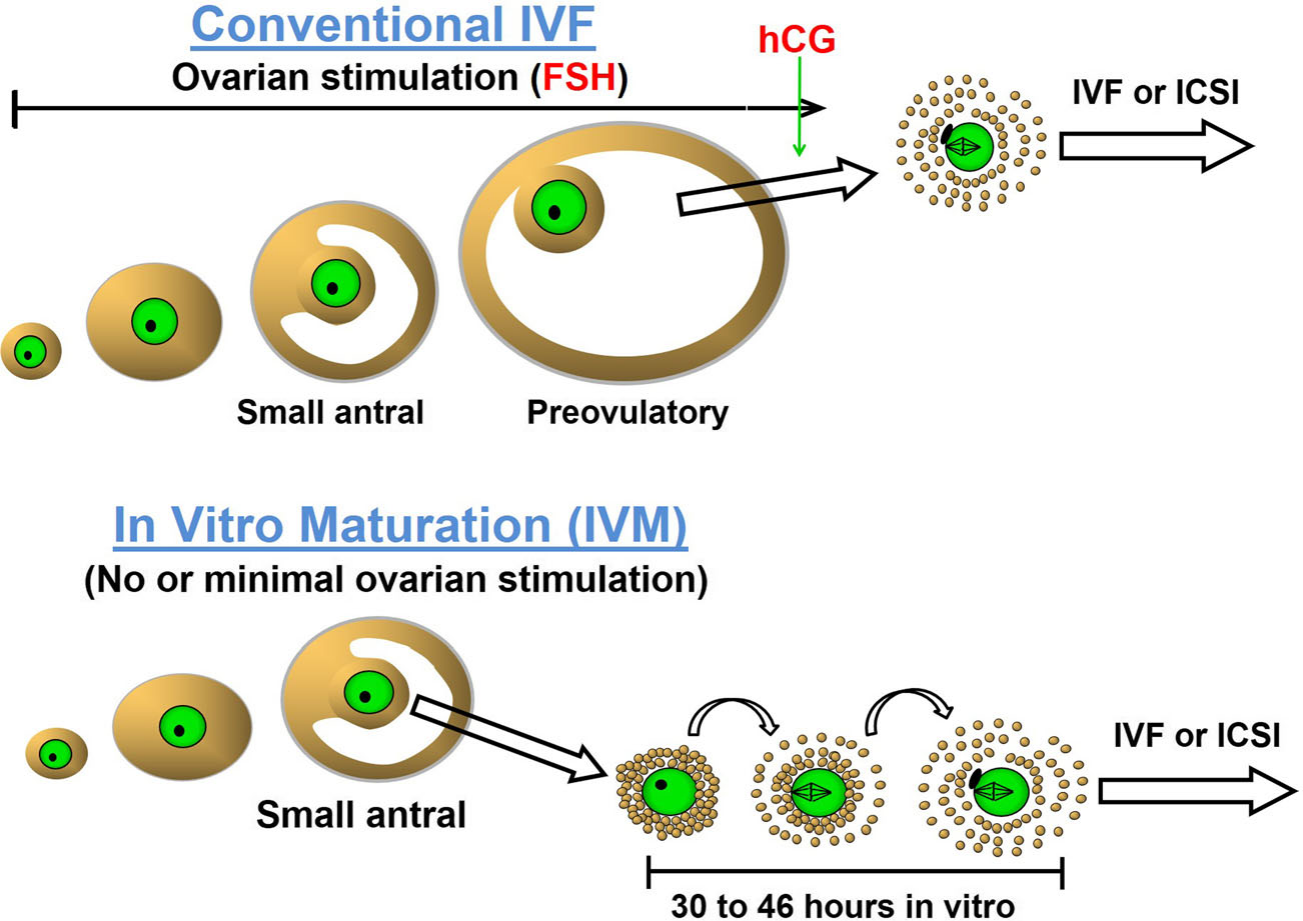
Differences between conventional IVF and IVM. The principal differences between conventional IVF and IVM are that in IVM cycles, patients receive minimal or no ovarian stimulation prior to OPU, oocytes are collected from small-medium sized antral follicles, and oocytes are meiotically matured in vitro from the germinal vesicle (GV) to metaphase II (MII) stage. Thereafter, mature IVM oocytes are treated exactly as per mature oocytes from conventional IVF. Adapted from [25]

The central challenge facing the IVM field was the discovery that IVM oocytes displayed reduced developmental competence compared to their in vivo matured counterparts. This held true even for IVM in agricultural animals, where IVM is used routinely for in vitro embryo production and has been studied far more extensively than in humans [24]. In all mammals, the follicular origin of the oocyte has a major impact on its subsequent development potential [25, 26] (Fig. 2). It appears that once the oocyte is removed from its follicle, its developmental potential is curtailed [26], which was recognized early on as an obstacle that would have to be overcome for IVM to succeed. During follicular development, few antral follicles gain dominant follicle status, most being subordinate and likely in varying states of atresia; heterogeneity of oocytes retrieved for IVM is thus due to their derivation from follicles at varying stages of development. Despite this, most oocytes from most species resume meiosis spontaneously once freed from follicles; human oocytes do this too although at a notably lower frequency than most mammals [8]. Although IVM oocytes are able to complete nuclear maturation, their ability to be fertilized and support subsequent embryo development is reliant on the inherent developmental competence of the oocyte acquired in vivo (traditionally called cytoplasmic maturation). Mammalian oocytes are largely transcriptionally quiescent during meiotic maturation, depending on processing of stored transcripts for protein synthesis and post-translational mechanisms to complete maturation, and acquire the competence to support early embryo development before embryo zygote genome activation [27]. Other aspects of cytoplasmic maturation including organelle redistribution, epigenetic and membrane modifications [28], are essential for fertilization and embryo development to proceed. Loss of synchrony between nuclear and cytoplasmic maturation, a common occurrence under in vitro conditions, is attributable to precocious meiotic resumption in vitro of an oocyte which was still acquiring developmental competence in vivo. This factor constitutes a major obstacle to the goal of generating high quality blastocysts from IVM oocytes.

**Fig. 3:**
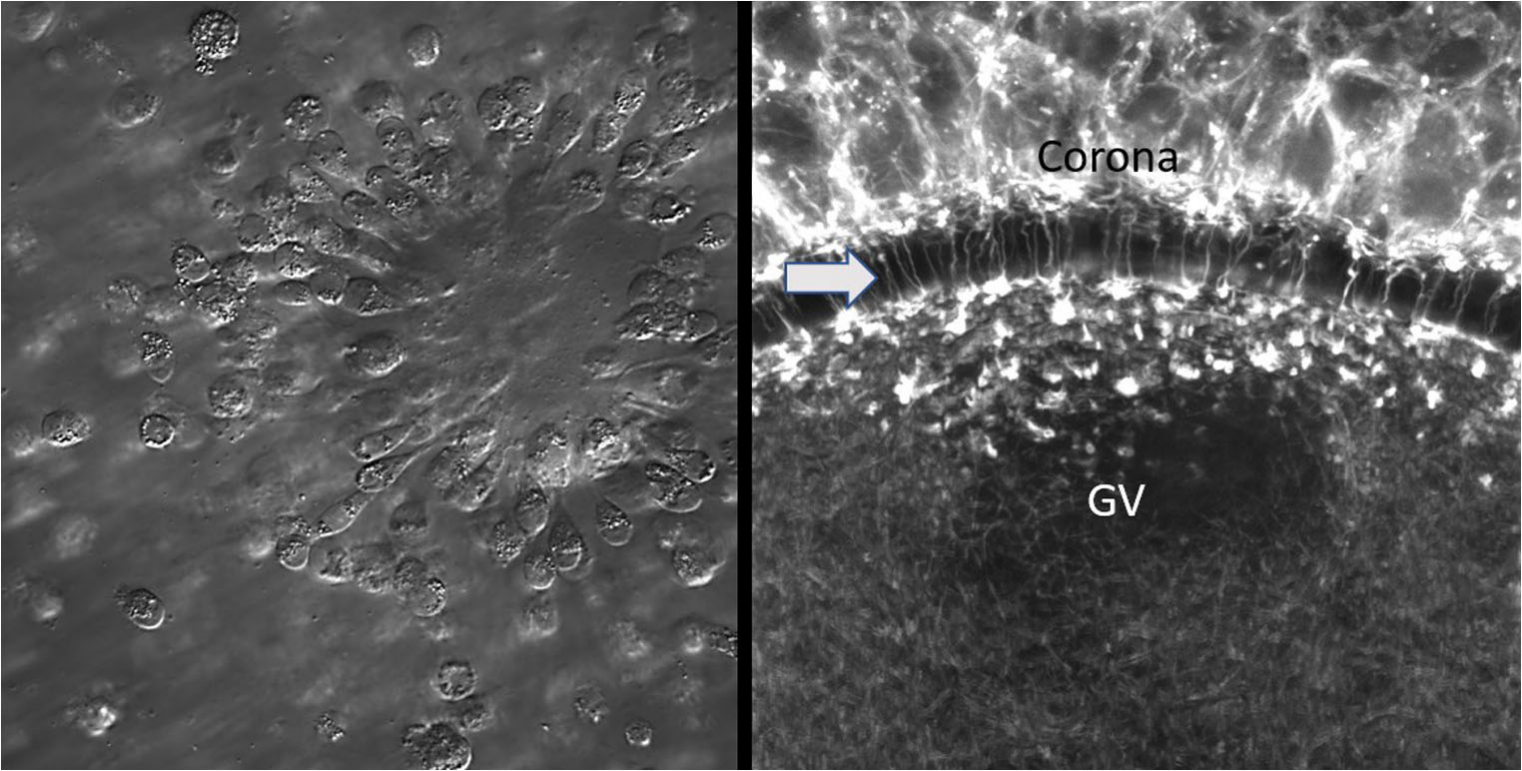
Oocyte–cumulus cell communication is fundamental to IVM success. Left, image of human MII oocyte collected after conventional ovarian stimulation and prior to removal of cumulus cells; note extensions from corona cells towards the oocyte surface. Confocal projection on the right is of an immature germinal vesicle (GV) stage bovine oocyte illustrating compact corona cells (top) sending numerous transzonal projections (arrow) that terminate on the oocyte cell surface. Alexa 555-phalloidin was used to label actin filaments in confocal image

Extraction from the follicular environment is inevitable for oocyte IVM and with it, the loss of all somatic cell and follicular fluid influences. However, several approaches can be taken to mitigate the impact of in vitro culture on the oocyte. The first is to try to mimic in vitro, as far as is possible, the in vivo follicular environment for the oocyte including retention of the architectural integrity of the COC and maintenance of meiotic arrest [29] (Fig. 3). Follicles prevent oocyte meiotic resumption by maintaining high levels of intra-oocyte cAMP through the infusion of natriuretic peptides and cGMP from the follicular somatic cells to the oocyte, as demonstrated in mouse and cow oocytes [30–33]. Hence, meiotic resumption can be readily prevented, and the COC structure retained, when oocytes from most mammalian species are cultured in the presence of natriuretic peptides (NPPC) or cAMP hydrolysis inhibitors (e.g., IBMX) [34]. The second approach is to include growth factors present in the follicular environment, including those like the EGF-like peptides, that are physiologically upregulated during oocyte maturation in vivo [21]. Notably, IVM media formulations have not changed substantially for decades. COCs, or naked oocytes, are cultured in various media supplemented with protein sources (serum or albumin) and gonadotropins. Most growth factors present in the follicular environment are not utilized in current standard IVM systems. As suggested by recent mouse and cow oocyte studies [35, 36], addition of growth factors into the IVM medium may yield oocytes with improved quality. These media modifications will continue to follow the findings from future research regarding the coordination of somatic cell–oocyte interactions.

## Laboratory and clinical approaches to IVM

**Fig. 4:**
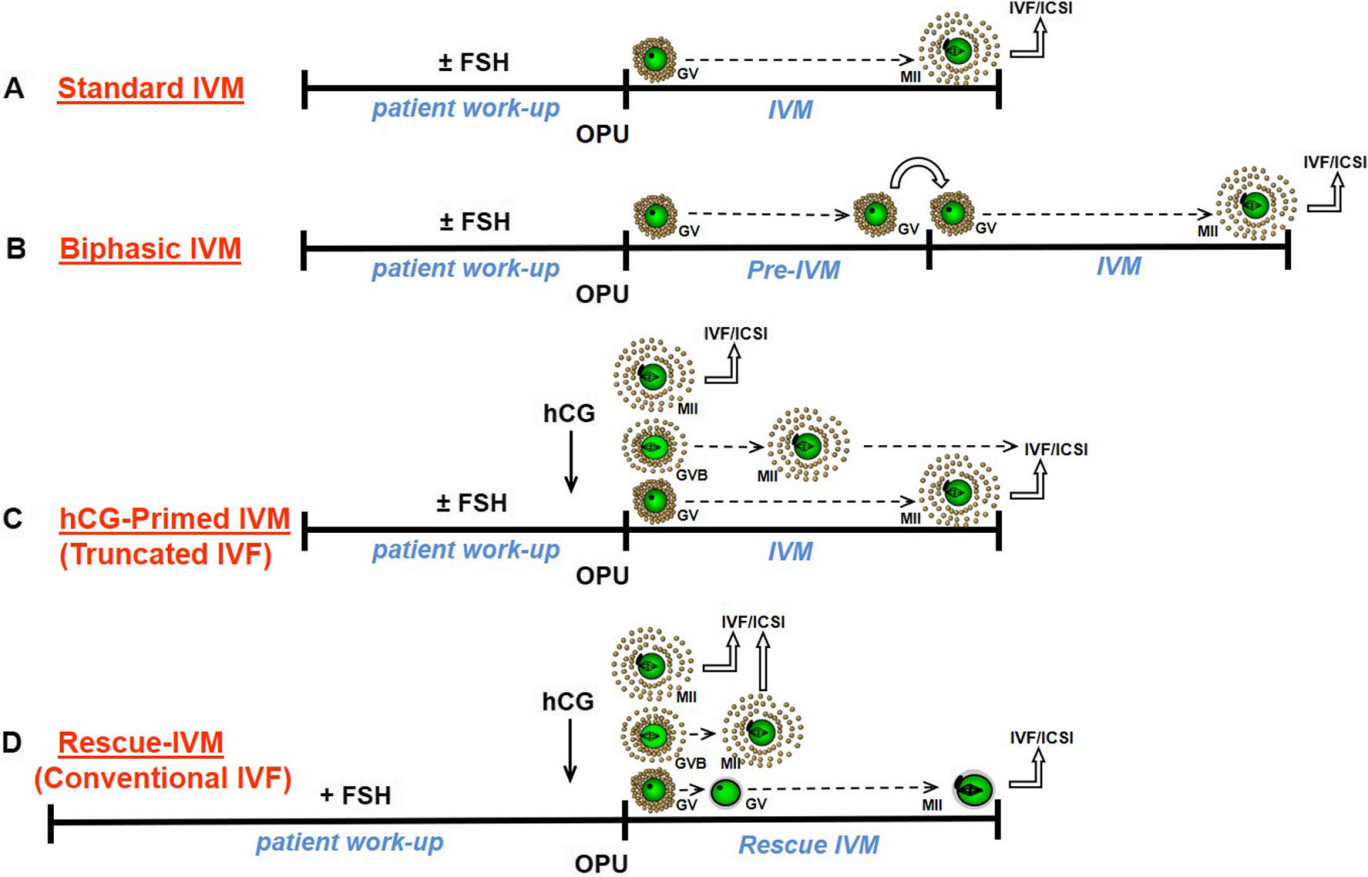
Major IVM protocols. **A** The original IVM protocol [8], where immature, GV-stage COCs are matured in vitro in one step to MII. Patients may or may not receive prior FSH priming as in either case all oocytes are at the GV stage at OPU. **B** A biphasic IVM protocol is a small variation on standard IVM, the notable difference being the additional pre-IVM culture step. Here, meiosis of immature cumulus-enclosed oocytes is deliberately arrested for ~ 24 h, before moving COCs into a meiosis promoting medium. Examples include the SPOM- and CAPA-IVM protocols. Patients may receive prior FSH priming, but not hCG priming, as the latter is incompatible with the need for intact compact COCs in this platform. **C** Patients receive a bolus of hCG prior to OPU, +/− prior FSH priming. A proportion (~ 10–20%) of oocytes are collected at the MII stage, some resume meiosis in vivo but are not mature (germinal vesicle breakdown (GVB) or MI), and the majority of oocytes are at the GV stage. The different stages of meiosis at OPU necessitate differing treatment in the laboratory: MII require fertilization on the day of OPU, whereas the maturing and immature oocytes require IVM culture. **D** This is the maturation in vitro of immature GV-stage oocytes collected from conventional IVF cycles after OS and ovulation triggering, mostly with hCG [38]. These are commonly regarded as medically unusable oocytes and are usually discarded in most IVF clinics [39]. These oocytes are usually naked, as oocytes are denuded of cumulus cells after OPU prior to ICSI; hence, rescue IVM oocytes are invariably cultured in a denuded state from the GV to MII stage in vitro

The introduction of a variety of clinical and laboratory approaches to IVM has led to considerable confusion and debate in the literature [18]. However, the traditional and broadly accepted definition of IVM [37], as originally described by Edwards [8], is the maturation of immature cumulus–oocyte complexes (COCs) collected from antral follicles that progress from the germinal vesicle (GV) stage through to MII in vitro over a species-specific time period (Fig. 4A). Variations on IVM theme are principally based on the administration of gonadotropins to the patient with the aim of obtaining oocytes with the highest developmental potential from larger antral follicles (stimulation with FSH) and/or to enhance the proportion of MII oocytes (through administration of a bolus of hCG before oocyte retrieval).

In simple terms, the laboratory component of IVM involves collection and culture of intact COCs over a time course expected to yield MII oocytes. Thereafter, mature oocytes and resultant embryos are treated exactly as they would be in a conventional IVF cycle. COCs are typically cultured in complex tissue culture-like medium with supplementation of a protein source and hormones (e.g., FSH +/− hCG), for 30–48 h, usually under atmospheric oxygen. In this review, we will not review the myriad of hormone, growth factor, and other additives that have been tested in IVM media. However, it is worth noting that there are three main recognized clinical IVM laboratory protocols [37], the choice of which, to some extent, is dictated by the clinical preparation of the patient prior to oocyte collection (Fig. 4A–C). Rescue IVM of GV oocytes from conventional IVF cycles is an additional consideration [38] (Fig. 4D), although it is not considered a clinical IVM procedure as it is a non-recommended and unconventional clinical practice [39, 40]. Table 1 briefly outlines the pros and cons of these different approaches to IVM.

**Table 1:** Advantages and disadvantages of the major IVM protocols

	Advantages	Disadvantages
**Standard IVM**	• Simple one-step culture system [8, 18] • All oocytes at the same immature meiotic stage at the start of culture [8, 41]	• Relatively low MII rates at ~ 50% [41–43] • Modest embryo yield and live birth rates [41, 43, 44]
**Biphasic IVM**	• Relatively high MII rates at ~ 70% [22] • Good embryo yield and live birth rates [22, 23, 43]	• Additional laboratory burden due the extra day of culture [22] • Has only recently been introduced in a limited number of IVM labs [23, 45]
**hCG-primed IVM**	• Relatively high MII rates at ~ 70% [46]	• Oocytes are collected at a mixture of meiotic stages [47] • Additional laboratory burden due to at least two rounds of ICSI per OPU [18] • IVM of GVB oocyte cohort is suboptimal as the exact extent of their meiotic progression at start cannot be determined [47] • Modest live birth rates [41, 47] • Prohibitive to the use of any pre-IVM culture approach [34]
**Rescue IVM**	• GV oocytes are common in conventional IVF patients • May generate additional embryos for transfer [38]	• Oocytes commonly have meiotic defects [48] • IVM in the absence of cumulus cell support → poor oocyte quality [20, 27, 40] • Questionable safety [39, 40]

### The role of FSH priming

Ovarian stimulation (OS) before oocyte retrieval in IVM has been applied with different protocols, including the use of clomiphene citrate, letrozole, or recombinant or urinary FSH. The most common OS protocol used and studied for IVM is a short course of FSH administered to the patient (FSH priming). FSH priming does not induce oocyte meiotic resumption in vivo, so this protocol always yields immature compact COCs after OPU (Fig. 4A, B). Based on extensive animal studies, FSH priming enhances follicular development and the meiotic and developmental competence of immature oocytes in vivo [49, 50]. Nevertheless, when priming with FSH results in follicle growth to a diameter of ~ 10 mm or more, the recovery rate of COCs from these follicles using a single lumen needle may be reduced [51]. Although the use of FSH priming in IVM cycles is based on solid rationale from animal studies and is quite common in clinical practice, there is no strong evidence from clinical trials for its use in human IVM [14, 41].

The first study suggesting that mild stimulation with FSH may improve oocyte yield and maturation rates was reported by Wynn et al. [52]. In this study, a course of 600 IU was used for 5 days, starting from day 2 of the cycle. A small RCT in 28 women with PCOS showed that a course of recombinant FSH for 3 days (150 IU daily from day 3 of the cycle), improved oocyte MII competence and implantation rate of cleaved embryos [42]. Nevertheless, FSH priming did not result in better clinical outcomes in women who did not have PCOS [14, 41]. There has been no consensus on the dose and duration of FSH priming for IVM, yet the common dosage is 150 IU of FSH daily for 2 or 3 days, starting from day 2 or 3 of the cycle or after a progestin withdrawal bleed [23, 53, 54]. Data from a small RCT by Mikkelsen et al. [55] showed no difference in apoptosis of granulosa cells and no difference in developmental competence of oocytes obtained, when oocyte retrieval was done 3 days, compared with 2 days, after the last FSH injection.

### Recapitulating the follicle environment: the evolution of biphasic IVM

Perhaps the most significant development in clinical IVM in recent years (Fig. 1) is the introduction of biphasic IVM (also called pre-IVM) into clinical practice (Fig. 4B). Although this represents a significant new direction for human IVM, the concept of pre-IVM has been in the animal literature for decades [56–58]. The basic principles of biphasic IVM culture systems are to (a) maintain the oocyte in vitro in a meiotically arrested (GV) stage, (b) retain intact the physical contact and paracrine signaling system of communication between oocyte and cumulus cells (Fig. 3), (c) foster and maintain an environment that allows the acquisition of developmental competence for the oocyte over ~ 24 h in the pre-IVM step, and (d) elicit resumption of and progression of meiosis under conditions mimicking the post-LH surge follicular environment (Fig. 4B) (reviewed in [34]). Refinement of these concepts eventually led to the breakthrough application of biphasic IVM to human oocytes in an IVM center in Belgium [22]. This proof-of-principle study in 30 oocyte donors represents a seminal contribution to the clinical IVM field [22], as it demonstrated the effectiveness of NPPC in human IVM, it demonstrated that biphasic IVM is superior to standard IVM, and it established the media formulation for “capacitation-IVM” (CAPA-IVM) which has been used in subsequent clinical trials. This study was followed by a safety study [59] and pilot RCTs in an IVM center of expertise in Vietnam of standard IVM versus biphasic IVM with favorable clinical outcomes for the biphasic approach [43, 60]. This culminated in a large RCT comparing the efficacy of biphasic IVM with conventional IVF, in which patients in the IVM arm received 150 IU of hMG daily for just 2 days, which was 5.5-fold less FSH than patients received in the IVF arm of the trial [23]. Although the difference in live birth rate after the first embryo transfer between biphasic IVM (35%) and conventional IVF (43%) was only 8% [23], which illustrates the potential of biphasic IVM to narrow the efficiency gap between IVM and IVF, the higher number of usable embryos after conventional IVF resulted in an almost 19% lower cumulative ongoing pregnancy rate at 12 months after randomization per started IVM cycle compared to conventional IVF.

### Triggering before OPU (hCG priming)

hCG and GnRHa have been used for triggering before oocyte retrieval in IVM, although most of the available data are with hCG triggering. The first successful application of hCG triggering before oocyte retrieval in IVM was reported in 1999 [61]. Since then, the use of hCG triggering in IVM has been adopted by many centers. Often hCG triggering in IVM is combined with a short course of FSH priming, except in the context of urgent fertility preservation [62]. Nevertheless, OS with FSH followed by a bolus of hCG may in many respects be considered a “truncated IVF” cycle [18] (Fig. 4C).

The hypothesis is that hCG may promote the initiation of oocyte maturation in vivo and the time course of oocyte maturation in vitro is hastened, and hence, the mature oocyte rate is increased. Therefore, it is possible that pregnancy rates may be improved by priming with hCG prior to immature oocyte retrieval [16]. The largest study of IVM using hCG triggering, combined with FSH priming, included 921 PCOS women. The oocyte maturation rate was 71% and the cumulative live birth rate over 12 months after one IVM cycle was 33.4% [46]. However, a Cochrane review recently found no conclusive evidence that hCG triggering before oocyte retrieval and IVM would have an effect on clinical pregnancy or live birth rates [63]. The authors provided low quality evidence suggesting that hCG priming may reduce clinical pregnancy rates, although these findings were limited by the small size of the data set. A recent RCT conducted on 172 cancer patients having IVM for fertility preservation verified the dubious role of ovulation triggering in IVM treatment. The authors found no difference in number of retrieved oocytes or the number of mature oocytes between the three interventions of no triggering, hCG triggering, or GnRH agonist triggering [64].

### Fresh transfer or freeze-only for IVM?

In IVM cycles, the follicular phase and duration of endometrial exposure to adequate estradiol levels are much shorter than in stimulated IVF/ICSI cycles. Yet, according to retrospective data, fresh embryo transfer (ET) was feasible for the majority of IVM treatment cycles after a short FSH-priming protocol for a least 3 days, with satisfactory pregnancy outcomes, either using a non-triggered IVM protocol [65] or hCG-triggered IVM protocol [66]. Nevertheless, based on the observation, again with retrospective data, that live birth rates after frozen ET (FET) were higher compared to fresh ET [53, 54], it was suggested that endometrial quality in non-hCG-triggered IVM cycles may be suboptimal for normal embryo implantation. In a prospective cohort study of 68 women who underwent endometrial sampling, an aberrant expression pattern of steroid receptors in endometrium in non-hCG-triggered IVM cycles was observed as well as a deficient mid-luteal histological signature of endometrial receptivity, possibly due to the combination of a short phase of endometrial proliferation and exposure of the endometrium to insufficient levels of progesterone [67]. Therefore, a freeze-only strategy was proposed for non-hCG IVM cycles [44]. The only RCT comparing live birth rates between fresh transfer or freeze-only strategies in non-hCG IVM cycles was published recently, which showed that frozen transfer provided a significantly higher live birth rate than fresh transfer [68]. In parallel, there is also emerging evidence that success rates after conventional OS and IVF in high responders [69] may be improved when a freeze-all approach is adopted instead of fresh embryo transfer. Although embryo cryopreservation has gained widespread acceptance, a recent concern in the approach of women with PCOS who undergo FET is the observation that FET may be associated with an increased risk of early pregnancy loss [70] and hypertensive disorders of pregnancy (HDP) [71], if FET is performed in hormonal replacement therapy (HRT) cycles. This is possibly due to inadequate progestin support [72]. In view of this, it seems mandatory to develop efficient clinical protocols for FET that are associated with a lower risk of adverse obstetric events in general and, in particular, in women with PCOS who undergo IVM.

## Selection of suitable patients for IVM

IVM of oocytes has been advocated as a safer alternative for conventional OS because of the ability to avoid the side effects and risks that are associated with OS in women with elevated functional ovarian reserve [12], including OHSS and ovarian torsion. In recent years, strategies have been developed to reduce OHSS risk, and the adoption of GnRH agonist triggering in combination with a policy of freeze-all embryos can eliminate the severe type of OHSS [73]. Therefore, clinicians have become less concerned about hyperresponse after OS. Furthermore, the observed relationship between high oocyte yield and favorable cumulative live birth rates per IVF cycle may be used as an argument by IVF practitioners to stimulate the ovaries of predicted high responders with higher doses than before the “freeze-all” era. Consequently, severe OHSS has been diminished, but moderate OHSS persists in many European countries [74] and in the USA [75]. Although the majority of predicted high responders accept the inherent risk of OS, a considerable proportion of women would embark on a less efficient fertility treatment if the burden of the treatment would be lower [76]. IVM could have an emerging role in this specific group of patients, although it is currently unknown to which extent women would accept a lower chance of pregnancy in return. Subfertile women with PCOS who are eligible for ART are probably the best candidates for IVM. PCOS is the most common endocrine condition in women and has an overall prevalence of approximately 10% according to the diagnostic criteria. These women may expect to have sufficiently high numbers of immature oocytes to make up for the inherently lower efficiency of IVM compared to conventional OS for IVF [77, 78]. Not only can women with PCOS exhibit excessive response to OS, a subset of them, especially those with hyperandrogenism and/or obesity, may have a narrow window of optimal ovarian response: OS in these patients requires frequent monitoring and may result in suboptimal outcome if hypo-response is observed [79]. For these patients, IVM may be an attractive option because monitoring of follicular growth can be kept to a minimum and oocyte retrieval for IVM can be scheduled at the patient’s convenience. In view of this, after patient counseling and discussion of pros and cons of conventional OS and IVM, a subset of patients with PCOS may embrace IVM as an alternative, simplified, and low-burden ART.

Anti-Müllerian hormone (AMH) correlates with the severity of the PCOS phenotype [80], with the highest AMH serum levels found in women with the classical PCOS phenotype A, characterized by PCO-like morphology of the ovaries, ovulatory dysfunction, and hyperandrogenism. Previous research has shown that AMH as a proxy of oocyte yield is a strong predictor of pregnancy after IVM [77]. In a retrospective cohort study encompassing 320 women with PCOS who underwent IVM, Mackens et al. [81] illustrated the importance of assigning a specific phenotype to women with PCOS, based on a combination of Rotterdam criteria. Indeed, after adjusting for potential confounders, the PCOS phenotype significantly correlated with cumulative live birth rate (CLBR) after IVM; patients with the classical PCOS phenotype A had the highest CLBR (40% per started cycle) [81]. Although no prospective studies have compared clinical outcome after IVM and conventional OS in women with PCOS type A, such a comparative study could be a valuable future endeavor.

## IVM for fertility preservation

Cryopreservation of embryos or oocytes after conventional OS is currently the most established technique for fertility preservation (FP) in women who have not recently received gonadotoxic treatment [82]. However, OS is not possible in prepubertal girls and may be contraindicated in women with estrogen receptor positive breast cancer, although FSH-induced hyperestradiolemia may be counteracted or avoided with adjuvant therapies like selective estrogen receptor modulators or aromatase inhibitors [83]. In these cases, IVM of oocytes harvested via transvaginal follicular aspiration or, alternatively, derived from extracorporeal ovarian tissue may be considered as suitable techniques for FP, although the inherently lower meiotic and developmental potential of IVM oocytes will inevitably mitigate the prospective chances of a live birth in cancer survivors who return to use their vitrified IVM oocytes, when compared to a similar number of oocytes that are cryopreserved after conventional ovarian stimulation.

Data from a center of expertise in IVM for FP illustrate the feasibility of transvaginal egg retrieval for IVM in the follicular or luteal phase in 248 breast cancer patients included in a FP program before neoadjuvant chemotherapy [62]. In this largest series of cancer patients undergoing IVM for FP so far, with a mean age of 31.5 ± 0.3 years, a mean number of 6.4 ± 0.3 mature oocytes were cryopreserved after IVM. The feasibility and safety of performing IVM in emergency settings has also been shown in women diagnosed with hematologic diseases [84]. In cancer patients, the administration of a bolus of hCG before oocyte retrieval has shown to not improve the total number of mature oocytes for cryopreservation [64]. The first livebirth following vitrification of in vitro matured oocytes harvested transvaginally demonstrates the utility of IVM in the overall strategy of female FP [85] (Fig. 1). Sonigo et al. [86] observed that antral follicle count and AMH values above 28 follicles and 3.9 ng/mL, 20 follicles and 3.7 ng/mL, and 19 follicles and 3.5 ng/mL were required to obtain at least 15, 10, or 8 cryopreserved oocytes, respectively, after transvaginal egg retrieval for standard IVM. Based on these data, the concept of double IVM, implying the repetition of IVM cycles even within a very short time frame (< 10 days), may emerge as a viable and safe option for increasing the number of mature eggs available for FP [87].

In order to expand the sources of cryopreserved material in cancer patients, IVM can be combined with oophorectomy or ovarian biopsies from the contralateral ovary for cryopreservation of ovarian cortex [88, 89]. Cumulus–oocytes complexes recovered during processing of extracorporeal ovarian tissue adds to the pool of available material [90–92]. Patients with high risk of malignant invasion of the ovary, such as borderline ovarian carcinoma [93], leukemia, neuroblastoma, and Burkitt lymphoma, may be most suitable for IVM of oocytes from excised ovarian tissue (OTO-IVM), given that ovarian tissue transplantation poses inherent risks for tumor reintroduction. However, OTO-IVM remains experimental because the long-term safety studies have yet to be conducted. Moreover, lower maturation rates were observed after OTO-IVM [91], not unexpectedly, due to the fact that COCs derived from non-selected antral follicles from patients of different ages are unlikely to have completed key stages of folliculogenesis and would not be well supported by standard IVM protocols. The biphasic IVM platform described above has already shown promising results for OTO-IVM [45].

## IVM for resistant ovary syndrome

Resistant ovary syndrome (ROS) is a rare endocrine condition characterized by hypergonadotropic anovulation (WHO group 3) and infertility. Patients often suffer from primary or secondary amenorrhea with timely and spontaneous onset of secondary sexual characteristics [94, 95]. Serum levels of FSH and LH are elevated, in spite of normal levels of AMH and normal antral follicle counts [96]. The pathophysiology of this syndrome relies on the inability of antral follicles to respond to both endogenous and exogenous FSH. Genetic or immunologic abnormalities may explain antral follicle unresponsiveness to FSH although the etiology remains often unexplained [97, 98]. Mutations with loss of function [99, 100] and polymorphisms of the FSH receptor [101, 102] have been described. For ROS patients, IVM is currently the only viable alternative option to egg donation, with several live births reported [103, 104].

## IVM for poor responders

Women exhibiting a poor response to exogenous gonadotropins have a reduced chance of achieving pregnancy, and the optimal management of these patients remains a matter of debate. Among the approaches considered, the practice of using high doses of gonadotropins in poor responders has not been supported by evidence [105]. Despite reports of livebirths obtained after rescue IVM [38, 106, 107] or unprimed immature oocyte retrieval [108], IVM as practiced yielded limited success in poor prognosis patients of either advanced reproductive age and/or low ovarian reserve. Indeed, the success of IVM relies heavily on the number of oocytes retrieved from a patient, even more than in a conventional OS cycle for IVF, due to the unpredictable recovery of COCs, suboptimal meiotic maturation rates from IVM of ~ 50% [109], and higher embryo attrition rate using IVM compared to conventional OS [110]. In addition, the fact that AFC, AMH, and total testosterone are the only independent predictors of oocyte yield in PCOS patients undergoing IVM [77] suggests that poor prognosis patients are not well suited to IVM.

## IVM in women with oocyte maturation defects

A small subset of infertile women exhibit oocyte meiotic maturation defects, in which immature (GV, M1 stages) oocytes are obtained after repeated conventional OS cycles [111]. These women have few alternatives available to them for infertility treatment. Some patients have underlying genetic defects in their oocytes [112] and IVM is unlikely to help their infertility. In other patients the pathophysiological basis for the disorder is unknown. The few case series deploying IVM for these patients have yielded disappointing results [103, 113]. Future developments with the IVM platform, including the use of biphasic IVM systems where meiosis is induced in vitro during the IVM phase (reviewed in [34]), could offer new opportunities for patients affected by such conditions.

## Safety aspects of IVM

All information and data available to date suggest that IVM is safe for both patients treated and children born from the technique [37, 114]. Indeed, for women with PCOS, IVM is less hazardous than treatment with conventional OS and IVF as the risk of OHSS is eliminated in IVM pregnancies [23, 54]. For some time, there were concerns about the higher rate of miscarriage in IVM cycles relative to conventional IVF [115], which is now know to be attributable to the use of fresh ET in IVM cycles [54], and hence with the adoption of a freeze-all strategy for IVM, miscarriage rates are the same as in conventional IVF [23]. In terms of pregnancy and obstetric complications, outcomes are not different for IVM and conventional IVF pregnancies in terms of rates of ectopic pregnancies, gestational diabetes, and antepartum hemorrhage [23, 114]. One study found a significant increase in hypertensive disorders of pregnancy in IVM compared to IVF pregnancies [116], but this was not found in the subsequent prospective RCT of biphasic IVM versus conventional IVF [23].

In terms of fetal and neonatal development, some concerns have been expressed about possible epigenetic risks for IVM children as oocyte meiosis occurs in vitro. The two studies to date that have examined the status of key imprinted genes in human IVM oocytes [59, 117] suggest that IVM does not interfere with genomic imprinting establishment. This is corroborated by imprinting studies using chorionic villus and cord blood samples from children born from IVM [118]. Consistent with findings from these epigenetic studies, recent conclusions from a meta-analysis [114] and from a RCT [23] found that the major measures of neonatal outcomes are not different between IVM and IVF babies, including preterm birth, spontaneous preterm birth, iatrogenic preterm birth, low or high birth weight, large for gestational age birth, congenital anomalies, and admission to the neonatal intensive care unit.

Follow-up studies of 2-year-old IVM children show normal growth and body weight compared to OS-IVF children, and there is no evidence of a delay in mental development in IVM children [119–121]. A long-term follow-up study of IVM children and adolescents up the age of 19 found no increased risk associated with IVM compared to IVF [122]. Collectively, these studies provide a degree of reassurance that outcomes for mothers and children born from IVM do not differ from conventional OS-IVF [114]; however, to date, IVM numbers remain low and ongoing follow-up studies of IVM children are warranted.

## Divergent regional perspectives on the role of IVM

Access to reproductive health care is excellent in European countries. Large numbers of reproductive medicine centers continue to contribute high level of safety and efficiency measures in current ART treatment options. Despite the linear relationship between cumulative birth rates and ovarian response after conventional OS and IVF/ICSI, even while the risk of iatrogenic complications and side effects has been reduced during the past decade, the perceived burden by patients of conventional OS remains high, especially in predicted high responders [76]. In women with excessive antral follicle counts, IVM has a better safety profile and may be advocated as a minimal-burden treatment option [123]. After balanced counseling of pros and cons of IVM compared to conventional OS, the tendency towards mild stimulation treatment with relatively lower success rates may appeal to a subset of high responders in countries where the out-of-pocket cost for the patient undergoing ART is relatively low (Table 2).

**Table 2:** Empirical analysis of factors that may modulate the uptake of IVM in different regions

Regions	Availability of reproductive care services	Out-of-pocket costs for patients	Incentive for IVM for infertility treatment	Incentive for IVM for onco-fertility preservation
Europe	+++	+	**Low incentive**: - Reduced efficiency compared to OS - Utilization of freeze-all strategies in high responders - Limited cost savings for the patient	**High incentive**: - Utilization will grow as more centers develop expertise in IVM - Focus on OTO-IVM in spite of experimental nature
USA and Canada	+++	+++	**Low incentive**: - High cost of ART for patients means prioritizing treatments with maximal efficiency	**High incentive**: - Utilization will increase as more centers develop expertise in IVM - Focus on OTO-IVM in spite of experimental nature
Russia	+++	++	**Low incentive**: - Relatively high cost of ART for patients means prioritizing treatments with maximal efficiency	**High incentive**: - Utilization will grow as more centers develop expertise in IVM - Focus on OTO-IVM in spite of experimental nature
Middle East and Maghreb	++	++	**High incentive**: - High incidence of patients with severe PCOS and underutilized safety measures in high responders - Limited uptake because of lack of experienced centers in the region and perceived complexity of clinical and laboratory IVM procedures	**Low incentive**: - Limited availability of onco-fertility programs in the region
India	+++	++	**High incentive**: - High incidence of patients with severe PCOS and underutilized safety measures in high responders - Limited uptake because of lack of experienced centers in the region and perceived complexity of clinical and laboratory IVM procedures	**High incentive**: - Utilization will grow as more centers develop expertise in IVM
Southeast Asia	++	++	**High incentive**: - High relative cost of gonadotropins - Increasing uptake in view of emerging number of centers in the region developing a high level of expertise in IVM	**High incentive**: - Utilization will grow as more centers develop expertise in IVM - Focus on OTO-IVM in spite of experimental nature
China	++	++	**Low incentive**: - High cost of ART for patients means prioritizing treatments with maximal efficiency	**Low incentive**: - Limited availability of onco-fertility programs in the region
Japan and South Korea	+++	+	**Low incentive**: - Reduced efficiency compared to OS - Utilization of freeze-all strategies in high responders - Limited cost savings for the patient	**High incentive**: - Utilization will grow as more centers develop expertise in IVM - Focus on OTO-IVM in spite of experimental nature
Australia and New Zealand	+++	+	**Low incentive**: - Reduced efficiency compared to OS - Utilization of freeze-all strategies in high responders - Limited cost savings for the patient	**High incentive**: - Utilization will grow as more centers develop expertise in IVM - Focus on OTO-IVM in spite of experimental nature
Central and South America	++	++	**Low incentive**: - Lack of experienced centers in the region and perceived complexity of clinical and laboratory IVM procedures	**Low incentive**: - Limited availability of onco-fertility programs in the region
Middle and Southern Africa	+	++	**Low incentive**: - Lack of experienced centers in the region and perceived complexity of clinical and laboratory IVM procedures	**Low incentive**: - Limited availability of onco-fertility programs in the region

The potential adoption of IVM in the ART clinics in Europe, and to a large extent in Australia and New Zealand, is in sharp contrast with prospects in the USA and Canada (Table 2). In North America, the projected uptake of IVM in ART clinics will be lower unless the efficiency of IVM culture systems can be markedly improved. ART practice patterns in the USA continue to follow ASRM guidelines in most clinics according to recent SART data. Historically, US clinics have been quick to adopt variations in protocols that increase marketing potential in a competitive atmosphere expected to be driven by capitalism. Hence, the rapid evolution of conventional OS strategies, freeze-all cycles, oocyte banking, and genetic testing approaches, all of which move into daily practice at a rate far in excess of more discriminating countries around the world. Given this backdrop, IVM has received little attention in the USA, admittedly in part because the latest advances in ovarian physiology that have formed the cornerstone of, for example, biphasic IVM, have yet to be fully appreciated. The much-needed change in attitude for the future will hopefully be prompted by this contribution to this special issue of *JARG*.

IVM was developed decades ago in South Korea and Japan [11], with many IVM programs continuing today. While the actual costs associated with one IVM cycle may be less than for one IVF cycle [124–126] in many Western countries, a major portion of IVF treatment or medication costs are often covered by public health and/or health insurance; therefore, IVM is not necessarily less expensive for infertile couples. Moreover, as long as IVM is less efficacious compared to conventional IVF, the cost for a baby using IVM may not be lower in many health care settings. In contrast, in countries with emerging economies, there is often no reimbursement system for infertility treatment. Therefore, IVM can be a more affordable ART lowering the out-of-pocket expenses for patients. In this sense, IVM could be even more attractive to infertile patients in lower income countries in SE Asia (Table 2). In fact, this is likely one of the principal drivers of the uptake of IVM in the many Asian ART centers with ongoing active IVM programs in China, Vietnam, India, Indonesia, Malaysia, and Thailand. By contrast, patients in South Korea and Japan are less likely to be motivated by the lower costs of IVM, even though they partly pay out-of-pocket for ART treatment, such that patients in these two countries have similar motivators to do IVM as patients in Europe and Australia.

## The need to develop a consortium of centers of expertise in IVM

As with any emerging ART, an anticipated barrier to clinical uptake of IVM will be access to clinical and laboratory knowhow of how to perform IVM. As IVM has been practiced for decades (but with low cycle numbers), there is in fact considerable literature on IVM. However, more recently, a major shortcoming of the human IVM literature is the enormous degree of confusion about what it is, and what it is not, and what constitutes clinically acceptable IVM practice [18], e.g., rescue IVM is very often conflated with standard IVM, when in fact rescue IVM has questionable safety and should probably not be practiced [37]. Consequently, many clinical practitioners are unclear about the current clinical status of IVM. A major gap for the field has been the need for a consensus statement on the clinical practice of IVM, something that in many respects has been addressed by the recent ASRM Committee Opinion on IVM declaring IVM non-experimental [37]. In addition, clinics are likely to be unsure how to implement an IVM practice. Importantly, IVM is substantially less technically demanding for embryologists to learn, compared to learning ICSI or embryo biopsy, and requires no additional laboratory equipment, although it does represent an extra procedure in the laboratory. Oocyte retrieval is more challenging in an IVM cycle compared to an IVF cycle; however, adaptations to standard retrieval technique enable any clinician to perform oocyte retrieval for IVM in most patients [127]. Moreover, clinical management and cycle monitoring of patients is greatly simplified compared to conventional OS or even ovulation induction cycles. Nonetheless, a misguided and insufficient understanding across the ART sector of what constitutes IVM, coupled with lack of sufficient centers of expertise that offer training in IVM, comprises current barriers to uptake and progress in IVM. In the past, pioneering clinical IVM hubs, such as those in Melbourne, Seoul, Osaka, Copenhagen, Monza, and Montreal, were instrumental in driving developments in IVM and accepting visitors from other clinics for training. These days the centers of expertise have shifted, to some extent, to Brussels, Paris, Tel Aviv, Ho Chi Minh City, and multiple locations in China including Guangzhou, Beijing, and Nanjing. It is imperative that these leading centers continue to pass on their expertise by offering training, including in the form of workshops, and that these are supported by industry and academic societies, such as the large and highly successful 2018 ASPIRE Masterclass in IVM, hosted by My Duc Hospital in Ho Chi Minh City, Vietnam. It is only through such concerted and collaborative effort by leading clinicians, scientists, academia and industry that IVM will obtain a foothold in routine ART practice, such that patients, in particular PCOS and cancer patients, will gain from the health and financial benefits that comes from infertility treatment with IVM.

## Gaps for future IVM research

1. *Improving embryo yield from IVM*: Despite recent improvements, a recognized need will be to improve blastocyst rates from IVM oocytes. This requires innovative and sophisticated new IVM culture systems, built on the latest scientific advances guided by continued animal oocyte biology research.
2. *Improving the recovery rate of oocytes in an IVM OPU*: The efficiency of IVM would be significantly enhanced if a higher proportion of oocytes could be recovered per follicle aspirated. This will require development of novel and sophisticated needle technology.
3. *New clinical approaches to patient management*: Can we further simplify patient management prior to IVM, such that cycle monitoring/management may not be needed at all? Further refinements on approaches such as random-start and the use of hormonal pretreatment are warranted.
4. *IVM as part of standard practice in fertility preservation*: IVM should be routinely integrated into the repertoire of technologies needed to maximize fertility preservation prospects for cancer patients. Research is needed on how to integrate the technology options of conventional OS with OPU-IVM, OTO-IVM, and ovarian tissue cryopreservation into clinical practice. In addition, studies integrating follicle in vitro growth technologies with pre-IVM are needed [128].
5. *IVM and planned oocyte cryopreservation*: Due to the convenient, lower cost, and mild stimulation nature of IVM, it provides an alternative for women seeking planned oocyte cryopreservation, especially younger women with a high AFC and AMH. To date, this approach has been poorly exploited and further research is needed.
6. *Towards zero-stimulation ART*: Furthering the development of sophisticated culture systems capable of supporting the growth and differentiation of oocytes prior to meiotic maturation, followed by IVM, will position the ART field to a point where minimal or zero stimulation is used.

## Summary points

- IVM of oocytes refers to the in vitro culture of cumulus-enclosed oocytes retrieved at the GV stage in medium that supports oocyte developmental potential.
- IVM is a non-experimental procedure as it has been practiced for several decades and the evidence to date suggests it is safe for women and offspring.
- IVM is a low intervention, mild approach to ART, well suited to patients with an excessive antral follicle count and is not suitable for poor prognosis patients.
- Although the technology is not new, IVM is not widely used, and incentives leading to enhanced uptake of its use in the ART clinic vary widely across the globe.
- With currently available IVM systems, clinical outcomes are lower than those after conventional ovarian stimulation in most women, but for some infertile women and after appropriate counseling, the improved safety and a simplified clinical approach will outweigh lower efficacy.
- Fertility preservation (FP) has enlarged the spectrum of fertility disrupting conditions, such as cancer, by offering a range of treatments that have evolved over the past decade. IVM has been and will continue to serve this population of patients in need of strategies to become parents precluded by conventional ARTs.
